# Post-traumatic Distal Femoral Salter-Harris Type II Fracture Unmasking an Incidental Osteochondroma as a Possible Stress Riser: A Case Report With Surgical Management

**DOI:** 10.7759/cureus.89364

**Published:** 2025-08-04

**Authors:** Bharat Sharma, Narendra Kumar, M C Shashidhar, Siddhartha Shankar Basumatary, Parag Garg

**Affiliations:** 1 Central Institute of Orthopaedics, Vardhman Mahavir Medical College and Safdarjung Hospital, New Delhi, IND

**Keywords:** distal femur, osteochondroma, pediatric orthopedics, salter-harris type ii fracture, stress riser

## Abstract

Osteochondromas are the most common benign bone tumors and are frequently discovered incidentally in the metaphyseal regions of long bones during growth. While typically asymptomatic, they may occasionally lead to complications such as neurovascular impingement, mechanical irritation, or pathological fractures. Salter-Harris type II fractures represent the most frequent physeal injuries in pediatric populations, particularly in rapidly growing regions like the distal femur. The simultaneous occurrence of a Salter-Harris type II fracture and an underlying osteochondroma at the same anatomical location is exceedingly rare. This report presents a unique case of a 15-year-old male who sustained a distal femoral physeal fracture following trauma, which revealed an incidental osteochondroma on imaging. Surgical management included open reduction and internal fixation using a distal femoral locking plate, along with excision of the osteochondroma. Histopathology confirmed a benign osteochondroma without features of malignant transformation. The case underscores the importance of comprehensive imaging in pediatric trauma, particularly when radiographs demonstrate atypical findings. The role of the osteochondroma as a biomechanical stress riser likely contributed to the fracture at this site. The dual surgical approach enabled both fracture stabilization and safe lesion excision. This case highlights the necessity of individualized, multidisciplinary management strategies in pediatric orthopedic trauma complicated by benign bone tumors and reinforces the value of considering occult skeletal anomalies in atypical fracture presentations.

## Introduction

Osteochondromas are the most common benign bone tumors, accounting for approximately 35-50% of all benign bone neoplasms [1]. These lesions typically arise in the metaphyseal regions of long bones during skeletal growth and are often discovered incidentally due to their asymptomatic nature. However, in rare scenarios, they may become clinically relevant due to complications such as neurovascular compression, mechanical irritation, or pathological fractures. Salter-Harris type II fractures are the most prevalent type of physeal injuries encountered in pediatric populations, particularly in rapidly growing bones such as the distal femur [2].

The coexistence of an osteochondroma and a Salter-Harris type II fracture at the same anatomical site is exceedingly rare, raising pertinent questions regarding the biomechanical implications of such lesions. Specifically, the role of osteochondroma as a biomechanical stress riser, capable of predisposing the bone to fracture under otherwise tolerable forces, is of significant clinical interest. In this case report, we present a unique case of a 15-year-old male who sustained a distal femoral physeal fracture that unmasked an underlying asymptomatic osteochondroma, highlighting diagnostic considerations and surgical management strategies.

## Case presentation

A 15-year-old male presented with pain and swelling in the right knee following a road traffic accident. There was no prior history of pain or swelling at the injury site, and no other swellings were present elsewhere in the body. On clinical examination, there was visible swelling, tenderness, deformity, and a palpable hard mass on the posteromedial aspect of the distal thigh.

**Figure 1:**
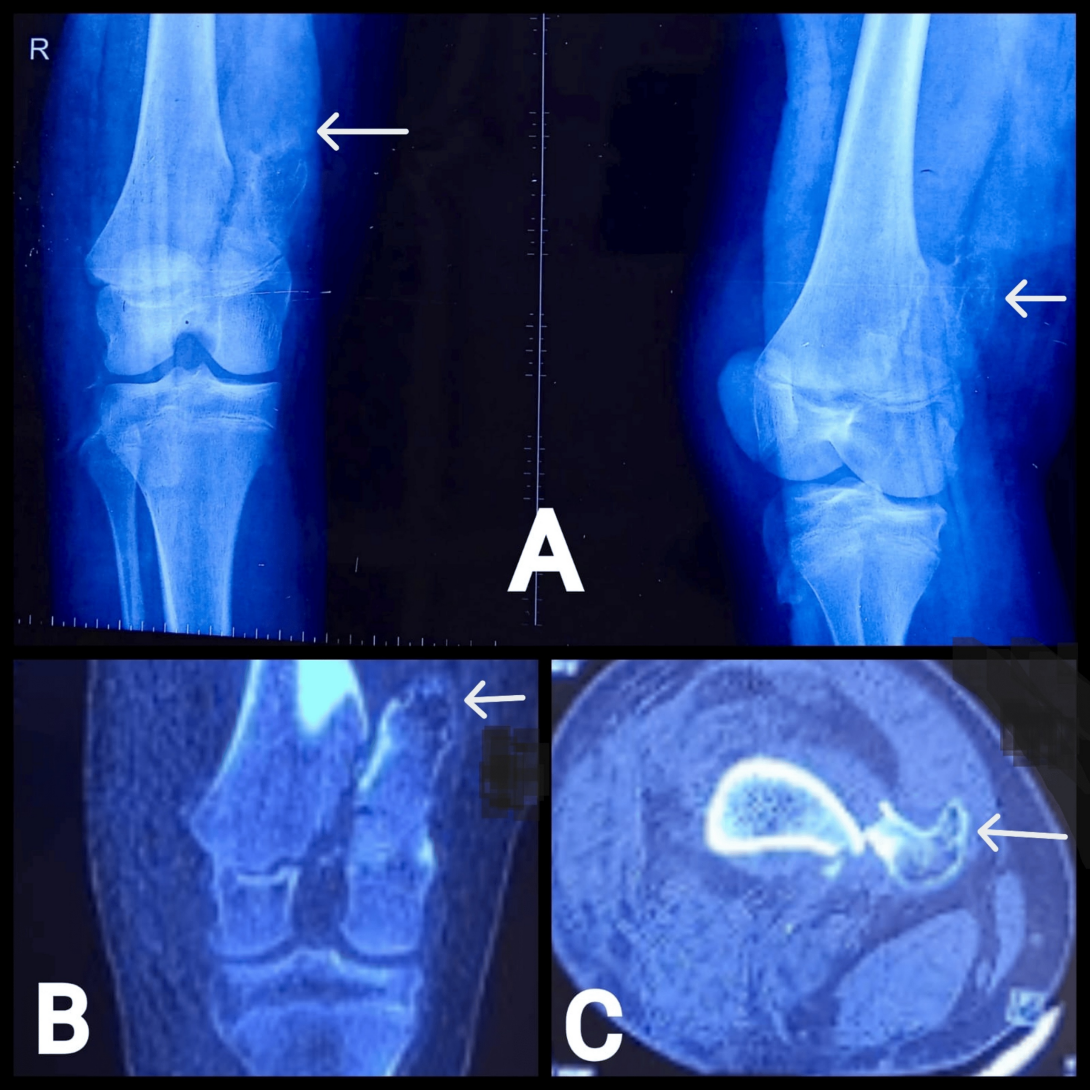
(A) Radiograph anteroposterior (AP)/lateral views, (B) CT scan (coronal cut), (C) CT scan (axial cut) showing osteochondroma with distal femur fracture.

Radiographs revealed a Salter-Harris type II fracture of the distal femur with a bony projection pointing away from the joint, suggesting an osteochondroma (Figure 1A). A non-contrast computed tomography (NCCT) scan confirmed the presence of an osteochondroma at the posteromedial aspect of the distal femur, with an intact stalk and no additional skeletal abnormalities (Figures 1B, 1C).

**Figure 2:**
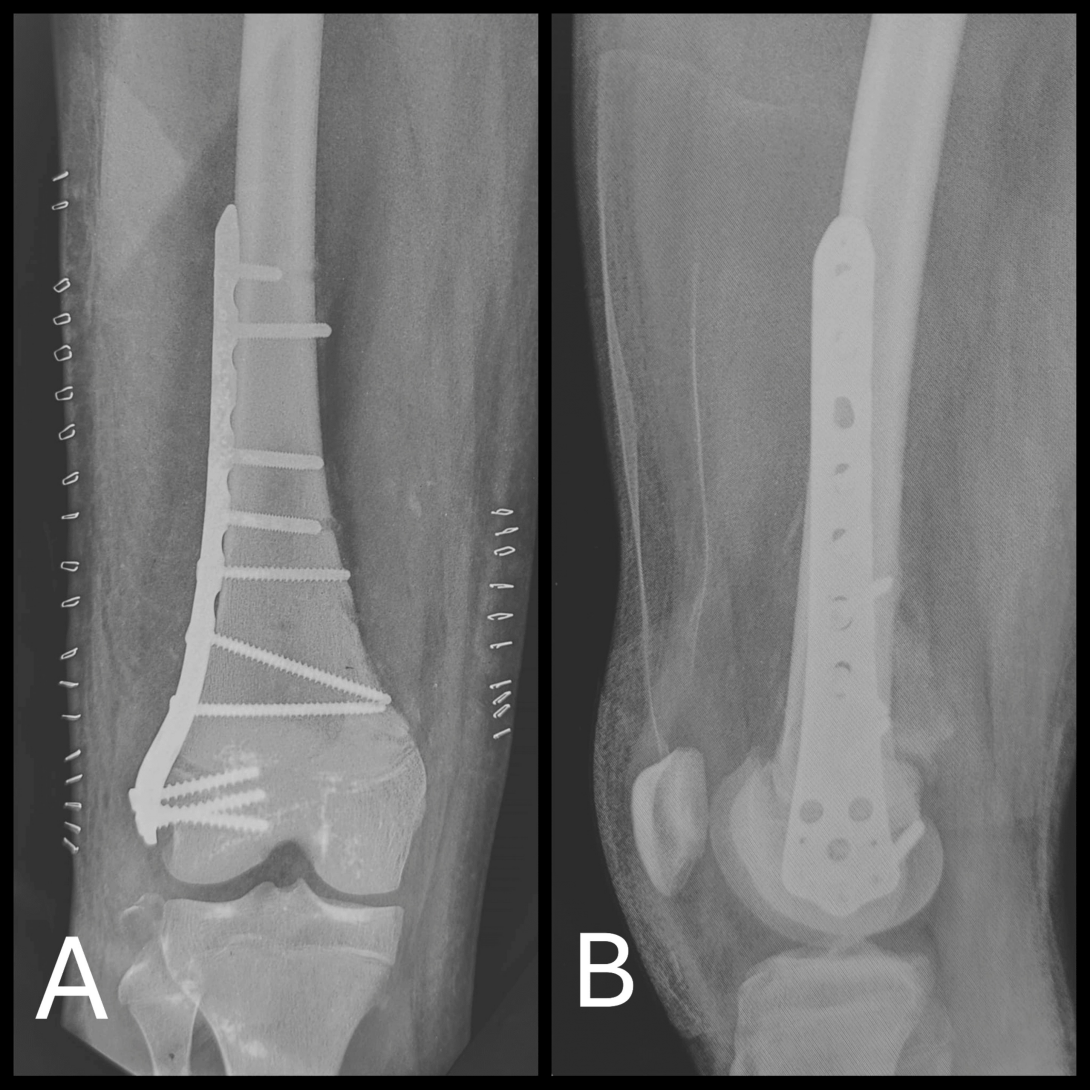
Post-op radiograph following osteochondroma excision and bony fixation. (A) Anteroposterior view; (B) lateral view

**Figure 3:**
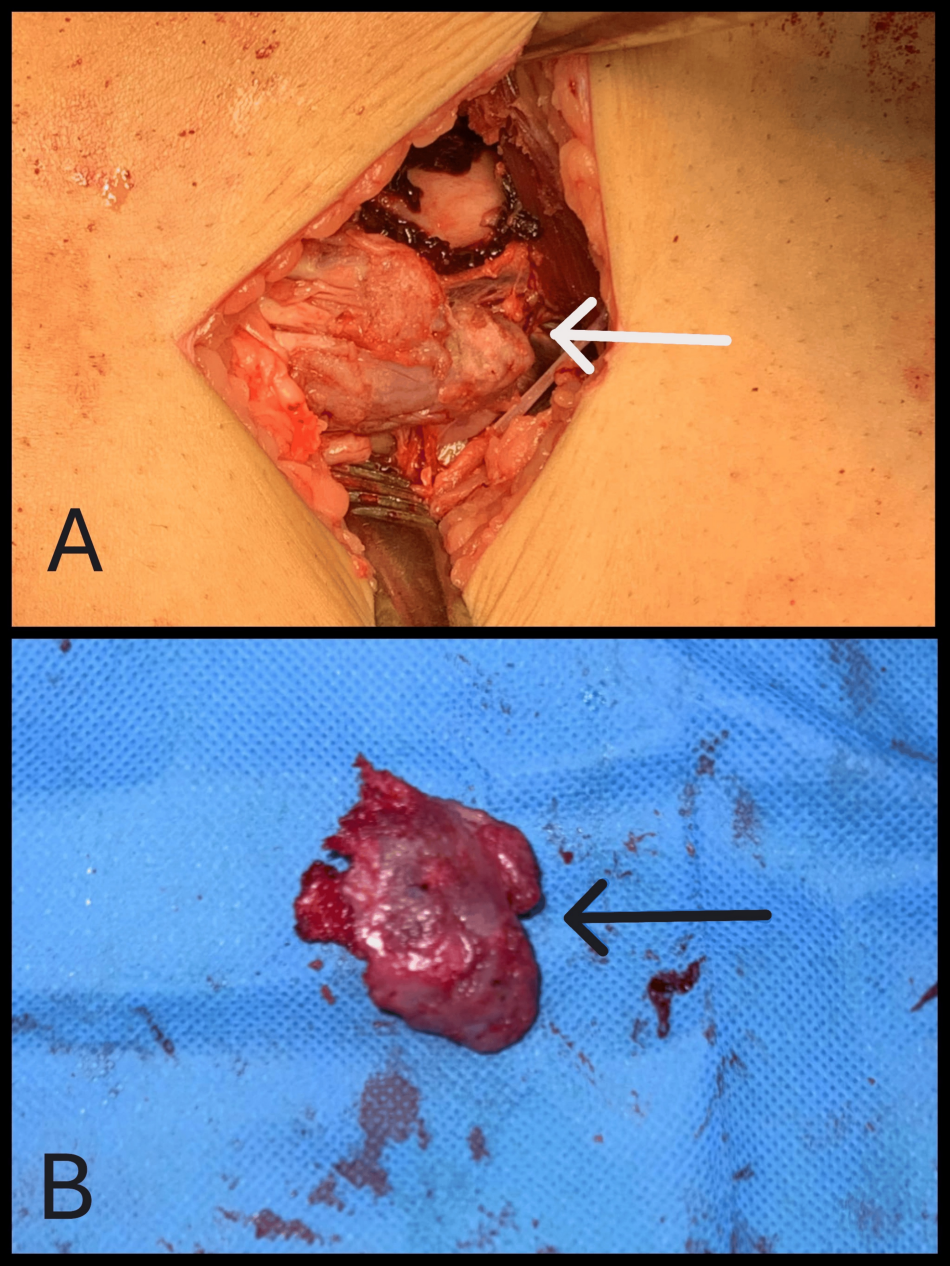
(A) Intra-op picture of osteochondroma; (B) osteochondroma after excision.

The patient underwent open reduction and internal fixation of the distal femoral fracture using a distal femoral locking plate via an anterolateral approach (Figure 2). Simultaneously, excision of the osteochondroma was performed through a medial approach using an extra-periosteal technique (Figure 3). The excised lesion was sent for histopathological examination. Postoperatively, the limb was immobilized in a slab, which was subsequently replaced with a hinged knee brace to allow for controlled mobilization.

**Figure 4:**
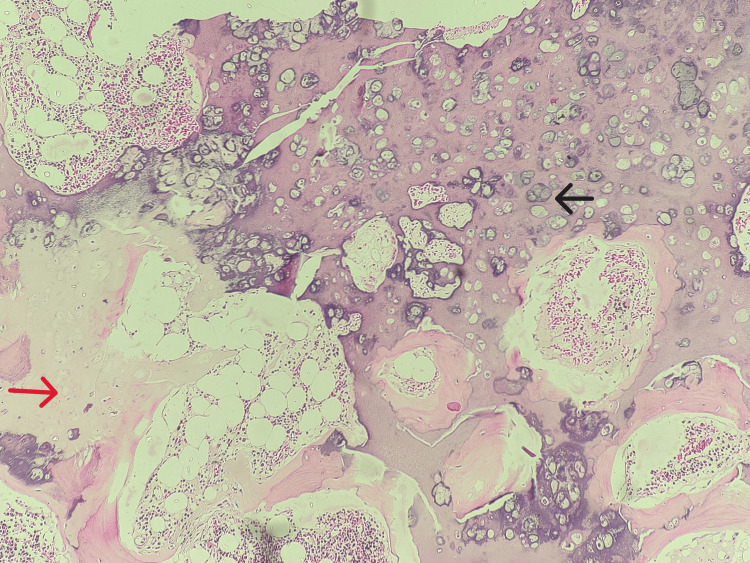
The photomicrograph reveals that chondrocytes are arranged in clusters and columns (black arrow) and endochondral ossification with trabecular bone containing normal marrow elements (red arrow).

Histopathological analysis revealed a cartilaginous cap composed of mature hyaline cartilage with benign chondrocytes arranged in clusters and individually scattered. Endochondral ossification was evident, and the underlying bone contained multiple trabeculae with normal marrow elements, including fat spaces. There was no evidence of atypia, nodularity, fibrous bands, or mucoid or cystic degeneration, confirming the benign nature of the osteochondroma and ruling out malignant transformation (Figure 4).

## Discussion

Osteochondroma is the most common benign bone tumor, typically presenting during the first two decades of life. It arises from the metaphysis of long bones and consists of a cartilage-capped bony projection continuous with the underlying cortex and medullary cavity. Although they are often asymptomatic, osteochondromas may become clinically evident due to mechanical irritation, neurovascular compression, or trauma. In rare instances, osteochondroma may be discovered incidentally during evaluation for a traumatic injury, as seen in this case [3,4].

Salter-Harris type II fractures involve the growth plate and metaphysis and are the most common types of physeal injuries in children and adolescents. The distal femoral physis is particularly vulnerable to injury due to its rapid growth rate [3,4]. The coexistence of a Salter-Harris type II fracture and an osteochondroma in the same anatomical region is exceedingly rare and raises questions about their temporal and causal relationship. In our patient, the osteochondroma was not previously symptomatic and was likely unrecognized until trauma led to acute clinical manifestation.

An important biomechanical consideration in this case is the role of the osteochondroma as a stress riser. Osteochondromas, due to their exophytic nature and alteration of normal bone geometry, can create localized areas of mechanical weakness or stress concentration. This can predispose the adjacent bone to fracture under forces that might otherwise be well tolerated by healthy bone [5,6]. In this context, the osteochondroma may have contributed to the initiation or propagation of the fracture site, effectively acting as a focal point of stress concentration [7].

Imaging plays a critical role in diagnosis. The classical radiographic appearance of an osteochondroma includes a sessile or pedunculated outgrowth projecting away from the adjacent joint. In our case, radiographs and CT imaging clearly demonstrated a posteromedial mass with cortical and medullary continuity, typical of an osteochondroma. MRI was not deemed necessary, as the lesion did not show signs of malignant transformation, such as a thickened cartilage cap or associated soft tissue mass [8].

Management of such cases should be individualized. The decision to excise the osteochondroma was influenced by its symptomatic presentation, proximity to the fracture site, and potential to hinder fracture healing or rehabilitation. The dual surgical approach allowed for fracture fixation and lesion excision to be performed simultaneously during the same procedure. Histopathological examination confirmed the benign nature of the lesion, with features consistent with a classic osteochondroma [9].

This case underscores the importance of thorough clinical and radiological evaluation in traumatic physeal injuries. The incidental finding of an osteochondroma at the fracture site highlights the necessity of a broad differential diagnosis when evaluating unusual physeal or metaphyseal appearances on imaging. Furthermore, it illustrates that trauma may unmask a previously asymptomatic lesion and that osteochondromas can act as biomechanical stress risers contributing to fracture risk, necessitating a multidisciplinary approach for optimal management [10].

## Conclusions

This case highlights the clinical importance of considering underlying skeletal anomalies in the evaluation of pediatric physeal injuries, particularly when radiographic findings are atypical. The identification of an incidental osteochondroma at the fracture site not only informed the surgical approach but also emphasized the lesion's potential contribution as a stress riser. Early recognition and comprehensive imaging, coupled with a multidisciplinary surgical plan, ensured optimal patient outcomes. Ultimately, this case underscores the value of individualized management in trauma cases complicated by benign bone tumors, reinforcing the need for a thorough and vigilant diagnostic process in pediatric orthopedics.
